# Uncovering the Salt Response of Soybean by Unraveling Its Wild and Cultivated Functional Genomes Using Tag Sequencing

**DOI:** 10.1371/journal.pone.0048819

**Published:** 2012-11-28

**Authors:** Zulfiqar Ali, Da Yong Zhang, Zhao Long Xu, Ling Xu, Jin Xin Yi, Xiao Lan He, Yi Hong Huang, Xiao Qing Liu, Asif Ali Khan, Richard M. Trethowan, Hong Xiang Ma

**Affiliations:** 1 Institute of Agro-Biotechnology, Jiangsu Academy of Agricultural Sciences, Nanjing, Jiangsu, China; 2 Department of Plant Breeding and Genetics, University of Agriculture, Faisalabad, Punjab, Pakistan; 3 Plant Breeding Institute, The University of Sydney, Sydney, New South Wales, Australia; Universidad Nacional Autonoma de Mexico, Instituto de Biotecnologia, Mexico

## Abstract

Soil salinity has very adverse effects on growth and yield of crop plants. Several salt tolerant wild accessions and cultivars are reported in soybean. Functional genomes of salt tolerant *Glycine soja* and a salt sensitive genotype of *Glycine max* were investigated to understand the mechanism of salt tolerance in soybean. For this purpose, four libraries were constructed for Tag sequencing on Illumina platform. We identify around 490 salt responsive genes which included a number of transcription factors, signaling proteins, translation factors and structural genes like transporters, multidrug resistance proteins, antiporters, chaperons, aquaporins etc. The gene expression levels and ratio of up/down-regulated genes was greater in tolerant plants. Translation related genes remained stable or showed slightly higher expression in tolerant plants under salinity stress. Further analyses of sequenced data and the annotations for gene ontology and pathways indicated that soybean adapts to salt stress through ABA biosynthesis and regulation of translation and signal transduction of structural genes. Manipulation of these pathways may mitigate the effect of salt stress thus enhancing salt tolerance.

## Introduction

Soil salinity is among the major environmental factors which significantly limits plant growth and yield. Previous studies reported increases in salinity tolerance of crop species; however the development of salt tolerant crop cultivars has been limited [1]. The complexity of the trait and lack of real urgency were reasons given for the lack of progress. The previous reports highlight the need for effective methodologies including genetic engineering if salt tolerance in a wide range of crops is to be enhanced [2]. The identification, evaluation and analysis of expression patterns of novel genes in response to abiotic stresses have been reported to provide a basis for effective engineering strategies to improve crop stress tolerance [3].

A large number of stress induced genes are classified into two major groups according to their putative function. The first group contains the genes encoding structural proteins, which are downstream effectors in the stress response pathway and include osmoregulatory genes [4], antioxidant proteins [5], aquaporins [6], late embryogenesis abundant (LEA) proteins [7], transporters/antiporters [8] etc. The second group comprises genes encoding regulatory proteins including transcription factors (TFs) and signal related protein kinases. The stress-related transcription factors play an important role in the regulation of salt and drought tolerance and generally include WRKY [9], [10], bZIP [9], MYB [11], [12], DREB [13], 9-cis-epoxycarotenoid deoxygenase (NCED) [14] and AP2/ERF proteins [15] etc. The protein kinases involved in signal transduction in response to different stresses include Ca^+2^ dependent protein kinases [16], mitogen-activated protein kinases (MAPKs) [17], receptor protein kinases (RPKs) [18], phosphatidylinositol kinase (PIK) [19], and serine/threonine protein kinase [20] etc. There are also extensive cross-links between responses to salinity, drought and other environmental and biotic stresses [15].

Any stress including salinity stress induces changes in gene expression, which cause a series of physiological and biochemical alterations. Several biochemical pathways such as photosynthesis and phenylpropanoid biosynthesis are significantly affected by stresses. These stresses change the normal function of other metabolic pathways including carotenoid biosynthesis, ABA biosynthesis and nitrogen fixation [9]. Despite significant progress during the past decade in developing understanding of pathways affected by salt stress, limited information is available on pathway dynamics in soybean under salt stress.

Of the strategies and techniques used to identify the novel genes, microarrays remain the most extensively used method in a range of crops including rice [21], *Arabidopsis* [22], *Brassica napus* [23], wheat [24], soybean [25] and tomato [9]. With the advent of next generation sequencing, recent reports compared the sensitivity, accuracy and reproducibility of some of the high through put techniques like serial analysis of gene expression (SAGE), microarrays, digital gene expression profiling (DGEP) etc. These reports suggest that DGEP with massive parallel sequencing achieved high sensitivity and reproducibility for transcriptome profiling [26], [27], [28]. Although DGEP lacks the ability to detect alternative splicing events compared to RNA-SEQ, it is much more affordable and clearly out-performed microarrays (Affymetrix) in detecting lower abundant transcripts [26] and novel genes.

This paper reports the genes and pathways affected by salinity stress that are likely involved in conferring salt tolerance in soybean based on information derived from DGEP data. For this purpose, the functional gene expression of a wild soybean genotype originating from the Chinese coast of the Yellow Sea was compared to a salt sensitive cultivated soybean in the presence and absence of salt stress. It is worth mentioning that those genes differentially expressed upon salt treatment between two different species, wild (salt tolerant) and cultivated (susceptible), will not necessarily correspond to ‘genes involved in conferring salt tolerance’, even if they were detected under high salt stress, because additional genetic differences not related to salt tolerance could be expressing under these conditions. Hence, these genes must be tested before considering them to confer salt tolerance.

## Results

### Response of wild and cultivated soybean to salinity stress

**Figure 1:**
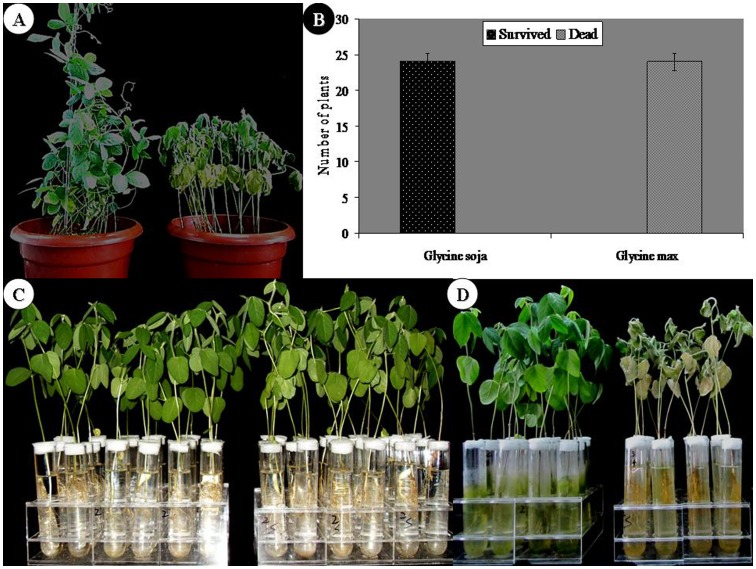
Response of wild and cultivated soybeans to NaCl stress. A, Response of salt tolerant genotype of *Glycine soja* (STGoGS) and salt sensitive genotype of *Glycine max* (SSGoGM) treated with 200 mM NaCl for seven days. B, Statistical comparison of two genotypes. C, Two sets of healthy plants of SSGoGM before NaCl stress application. D, Comparison of SSGoGM plants grown in the absence and presence of NaCl stress.

All plants of wild species survived while those of cultivated species died under 200 mM NaCl stress applied for seven days (Figure 1). The former is designated as salt tolerant genotype of *Glycine soja* (STGoGS) and the later as salt sensitive genotype of *Glycine max* (SSGoGM).

### Data generation and filtering

**Figure 2:**
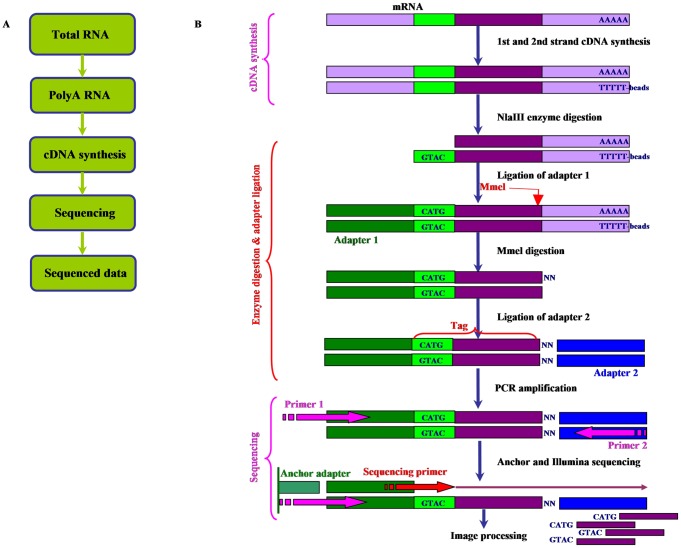
Digital gene expression profiling (DGEP). A, Outline of experimental process. B, Principle and procedure in detail; Beads of Oligo (dT) are used to enrich mRNA in the total RNA, and then are transferred into double-stranded cDNA through reverse transcription. Four base recognition enzyme *Nla*III is used to digest this cDNA, and Illumina adapter 1 is linked. *Mme*l is used to digest at 17 bp downstream of CATG site; Illumina adapter 2 is linked at 3′ end. Primer GX1 and Primer GX2 are added for PCR. Then, regain 85 bp strips through 6% TBE PAGE. The DNA was purified and followed by Solexa sequencing.

The Tag-seq protocol used [27] is similar to the LongSAGE approach [29], in which a restriction endonuclease NlaIII restricts each individual transcript in a sample and another type II restriction endonuclease (MmeI) generates a 21-bp tag restricting 17-bp downstream of NlaIII restriction site. The tags were cleaned and directly sequenced using massively parallel sequencing on the Illumina Genome Analyzer (see Materials and Methods; Figure 2).

The Tag-seq platform used four libraries generated from normal and salinity stressed root tissue samples of STGoGS and SSGoGM for comparison of their digital gene expression profiling (DGEP). The percentage of unambiguous tags was slightly higher in STGoGS (see Table S1). In total, normal and salinity stressed libraries contained clean tags of 3.375 and 3.331 million respectively in STGoGS, and 3.641 and 3.606 million respectively in SSGoGM (Table 1). Erroneous tags were removed to ensure high quality data. After filtration and removal of potentially erroneous tags, respective distinct clean tags of 126886 and 135790, and 236593 and 249119 remained. On average, 40.4 and 33.4% of distinct clean tags from STGoGS and SSGoGM respectively, mapped to genes. All the tags were mapped first to the whole soybean genome followed by closely related genomes. Those clean tags that could not be mapped to mRNA, mitochondria or chloroplast in the databases provided unique transcripts, without the pre-designed probes necessary with microarray. Unknown transcripts matched to distinct clean tags were 21% in both samples of STGoGS and 26 and 28% in control and salt treated samples of SSGoGM, respectively (Table 1, also see Table S1).

**Table 1:** Simple summary results of tag libraries and tag mapping to genes and genomes from salt tolerant genotype of *Glycine soja* (STGoGS) and the salt sensitive genotype of *Glycine max* (SSGoGM) in presence and absence of salt stress.

	Control	200 mM NaCl
**Total clean tags**		
STGoGS	3374902	3330577
SSGoGM	3641271	3606323
**Distinct clean tags after filtration from total clean tags**		
STGoGS	126886	135790
SSGoGM	236593	249119
**All distinct tags mapping to genes (Sense & anti-sense)**		
STGoGS	53167 (42%)	52711 (39%)
SSGoGM	82957 (35%)	79009 (32%)
**Unknown tags**		
STGoGS	26330 (21%)	28477 (21%)
SSGoGM	61168 (26%)	69688 (28%)

### Expression profiles of salt-responsive genes and verification by qPCR

Changes of gene expression in STGoGS and SSGoGM under salt stress were investigated using soybean Tag sequencing data generated via DGEP. A total of 1327 (826 up-regulated and 501 down-regulated) and 3627 (1709 up-regulated and 1918 down-regulated) genes were differentially expressed (DE) (FDR<0.001 and fold≥1) in STGoGS and SSGoGM respectively (Table 2).

**Table 2:** Summarized changes of gene expression in salt tolerant genotype of *Glycine soja* (STGoGS) and the salt sensitive genotype of *Glycine max* (SSGoGM) under salt stress.

	Upregulated	Downregulated	Total	Ratio
STGoGS	826	501	1327	1.6
SSGoGM	1709	1918	3627	0.9
**After removing redundancy**				
**Specifically expressed genes**				
STGoGS	275	215	490	1.3
SSGoGM	1082	1415	2497	0.8
**Commonly expressed genes**				
STGoGS	439	211	650	2.1
SSGoGM	403	247	650	1.6
**Expression pattern in SSGoGM of those from STGoGS**				
STGoGS	368	35		
SSGoGM	71	176		
Total	439	211		

A total of 3637 non-redundant salt-responsive genes were classified into different groups based on their expression patterns (Table 2, see for details of genes Table S2). Among these 490 (275 up-regulated and 215 down-regulated) genes were specifically differentially expressed (SDE) only in STGoGS and 2497 (1082 up-regulated and 1415 down-regulated) genes were SDE only in SSGoGM. In addition, 650 (439 up-regulated and 211 down-regulated in STGoGS, and 403 up-regulated and 247 down-regulated in SSGoGM) genes were DE in both species tested. Among the 439 up-regulated genes from STGoGS, 71 were down-regulated in SSGoGM while among 211 down-regulated genes from STGoGS 35 were up-regulated in SSGoGM.

**Figure 3:**
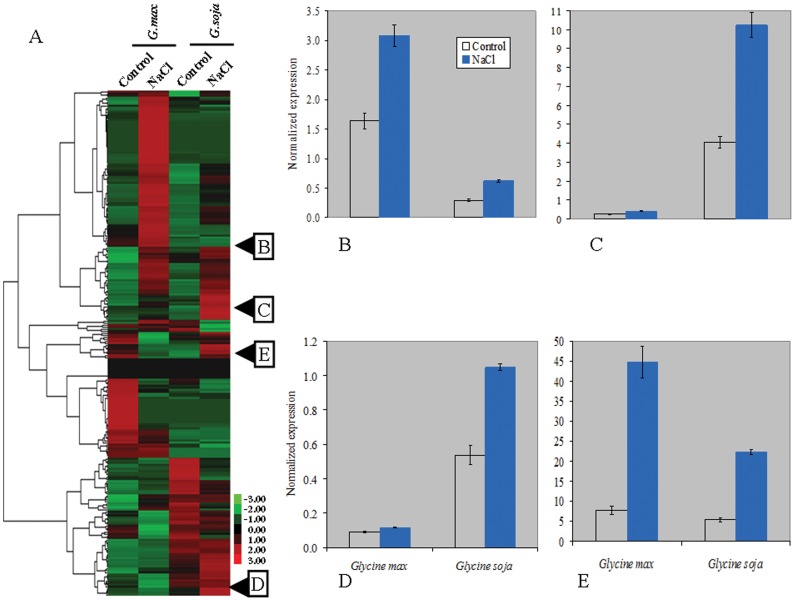
Normalized expression of differentially expressed transcription genes in salt tolerant genotype of *Glycine soja* (STGoGS) and salt sensitive genotype of *Glycine max* (SSGoGM) under 0 and 200 mM NaCl stress. A, hierarchical clustering of differentially expressed transcription genes based on their relative gene expression values (Z-score) computed using DGEP data. Red to green color indicates high to low expression levels. B to E, is relative expression based on qPCR data of GmMYB92, GmNAC2, GmWRKY and GmbZIP110, respectively while their position for the respective expression pattern in DGEP is also shown.

The qRT-PCR analysis of 20 randomly selected salt-responsive genes based on the DGEP analysis confirmed the DGEP results. The hierarchical cluster of TFs based on DGEP analysis (Figure 3A) and four selected TFs (Figure 3B to 3E) are presented for comparison of expression pattern between the two analyses/methods.

### Gene ontology (GO) enrichment analysis and Pathways annotation

Salt responsive genes were classified into different categories using gene ontology (GO) enrichment analysis and pathway annotation. All DEGs were mapped to GO-terms in the GO database to identify significantly enriched GO terms in DEGs compared to the genomic background. GO enrichment showed that a majority of GO molecular functions, biological processes and cellular processes were affected by salt stress in SSGoGM (Tables S3, S4, S5). However, the majority of GO-terms were common in both the species. Of 226 molecular functions, six were enriched in DEGs specifically expressed in STGoGS, 104 contained DEGs specifically expressed in SSGoGM and 116 GO functions contained DEGs common in both species. The GO enrichment process analysis identified 19 cellular processes enriched solely in DEGs from STGoGS, 159 enriched in DEGs specifically from SSGoGM and 217 enriched in common DEGs. Three cellular components were enriched in DEGs specific to STGoGS, 55 enriched in DEGs specific to SSGoGM and 62 enriched with genes affected by salt stress in both species (, S4, S5, also see Figures S1, S2, S3).

Pathway annotation helps to further understand the role of genes in biological pathways as different genes usually cooperate with each other to control biological functions. In order to assess the functional roles of salt responsive genes and to study the soybean's biochemical adaptations to salt stress, significantly enriched metabolic or signal transduction pathways in DEGs were identified via pathway enrichment analysis using the Kyoto Encyclopaedia of Genes and Genomes (KEGG); a public pathway-related database, and comparing this with the whole genome background. The identified pathways were either specific or common in STGoGS and SSGoGM.

**Figure 4:**
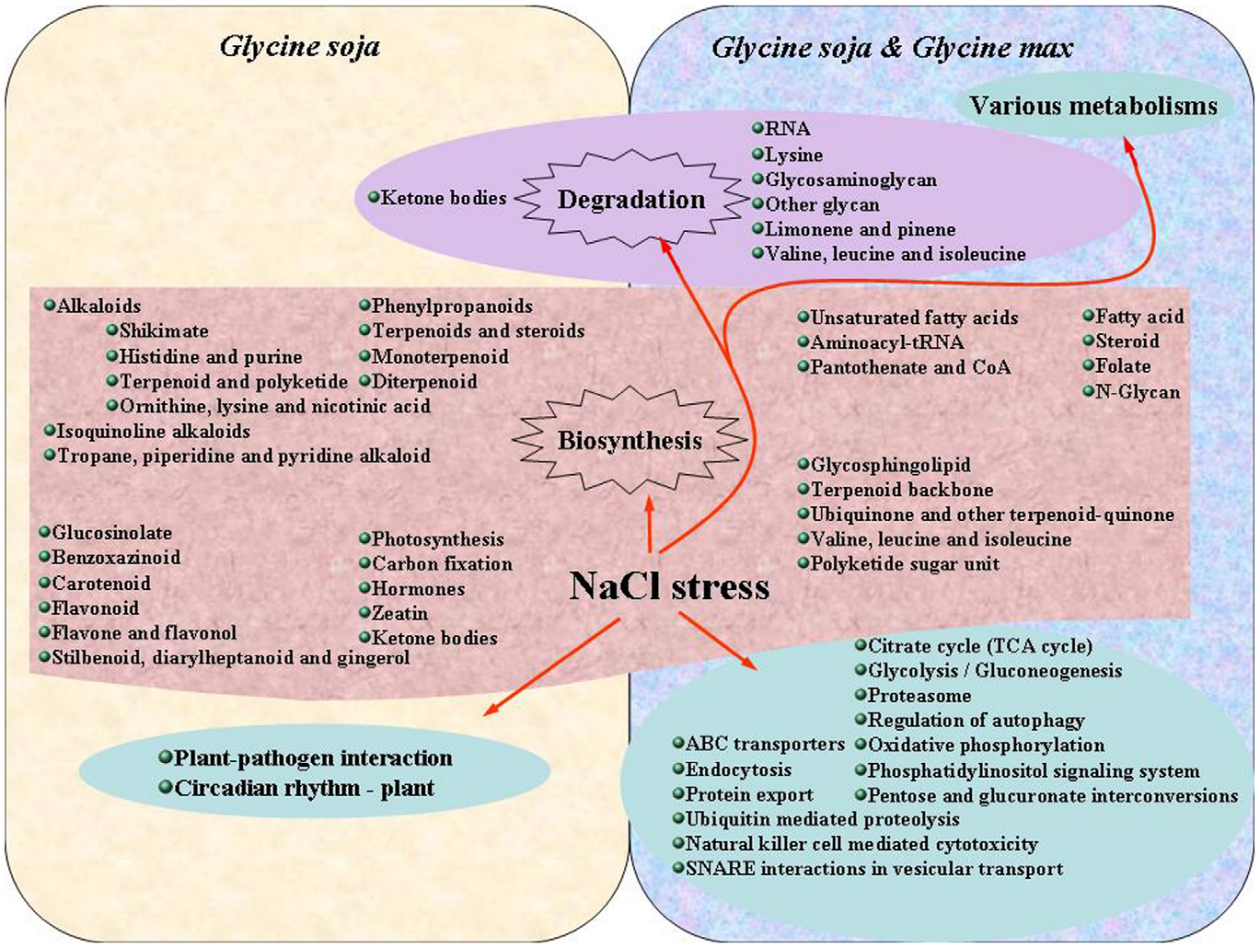
Pathways, annotated from KEGG database, enriched with differentially expressed genes specific to salt tolerant genotype of *Glycine soja* (STGoGS) and common in both.

Approximately 25 pathways were enriched in DEGs specific to STGoGS (Table S6). Prominent among them were phenylpropanoids biosynthesis (96 DEGs), plant-pathogen interaction (88 DEGs), and plant hormone biosynthesis (40 DEGs) (Figure 4, also see Table S6). Ninety five pathways were enriched in DEGs specific to SSGoGM. Eighty three pathways were enriched in DEGs common in both species. Prominent among these common pathways were metabolic pathways including methane and phenylalanine metabolism. Fatty acid and alpha-linolenic acid metabolism were enriched two-fold or more in DEGs from STGoGS compared to SSGoGM.

### Salt responsive transcription factors and signaling proteins

In response to salt stress in STGoGS, 19 specifically identified transcription factors (TFs) were classified into five major groups according to their putative DNA binding domains (Table 3, also see Table S7). The first group was the WRKY family which contained three genes (gnl|UG|Gma#S48315983, gnl|UG|Gma#S48315534, and gnl|UG|Gma#S39545382). All STGoGS specific WRKY family genes were up-regulated under salt stress. The second group contained four MYB family genes (gnl|UG|Gma#S39313776, gnl|UG|Gma#S48540161, gnl|UG|Gma#S47711991, and gnl|UG|Gma#S34273499). Only one gene (gnl|UG|Gma#S48540161) was down-regulated due to salt stress. The third group consisted of five NAC genes, three up-regulated (gnl|UG|Gma#S44358738, gnl|UG|Gma#S48313642, and gnl|UG|Gma#S48316644), and two down-regulated (gnl|UG|Gma#S48316686, and gnl|UG|Gma#S44358747). The fourth group contained one up-regulated gene (gnl|UG|Gma#S23068684) and one down-regulated gene (gnl|UG|Gma#S5143834) from basic leucine zipper (bZIP) family. The fifth group consisted of two up-regulated heat shock proteins (HSPs) (gnl|UG|Gma#S39318688, and gnl|UG|Gma#S48315341). The remaining transcription factors included Aux/IAA protein (gnl|UG|Gma#S4935632), RAV-like DNA-binding protein (gnl|UG|Gma#S26574424), and zinc finger (gnl|UG|Gma#S48315540) which were induced specifically in STGoGS. Only Aux/IAA protein (gnl|UG|Gma#S4935632) was down-regulated. Out of 48 transcription factors commonly induced by salt stress in both species, 7 belonged to WRKY, 10 to MYB, 5 to NAC, 4 to bZIP, 9 to HSPs, 4 to AP2/EREBP, and 3 to DREB gene families. Salt stress also changed the expression pattern of six other transcription factors. All commonly induced transcription factors were expressed similarly in both species except the AP2/EREBP gene (gnl|UG|Gma#S5126123) which was down-regulated in SSGoGM only. Further, all other genes were up-regulated in both species except three bZIP genes (gnl|UG|Gma#S34818024, gnl|UG|Gma#S34818016, and gnl|UG|Gma#S34818015) which were down-regulated in both species.

**Table 3:** Transcription factors (TFs) and signaling genes responsive to salinity stress in salt tolerant genotype of *Glycine soja* (STGoGS), salt sensitive genotype of *Glycine max* (SSGoGM) and common in both.

	STGoGS		SSGoGM		Common			
Gene	UR	DR	UR	DR	URS	DRS	URM	DRM
**Transcription factors**	**14**	**5**	**47**	**18**	**45**	**3**	**44**	**4**
WRKY family	3	0	2	0	7	0	7	0
MYB family	3	1	11	3	10	0	10	0
NAC family	3	2	4	0	5	0	5	0
bZip family	1	1	10	6	1	3	1	3
HSPs family	2	0	4	2	9	0	9	0
AP2 gene family	-	-	3	2	4	0	3	1
DREB gene family	-	-	2	1	3	0	3	0
Other TFs gene family	2	1	11	4	6	0	6	0
**Signaling genes**	**5**	**2**	**33**	**18**	**10**	**2**	**9**	**3**
**Stress related genes**	**7**	**7**	**12**	**14**	**27**	**10**	**24**	**13**
**Interesting unknown genes**	**24**	**19**	**-**	**-**	**32**	**20**	**20**	**32**
**Translation genes**	**2**	**0**	**4**	**7**	**2**	**0**	**2**	**0**

UR and DR is upregulated and downregulated respectively. URS and DRS, URM and DRM are respective upregulated and downregulated genes in STGoGS and SSGoGM respectively.

Seven signaling related genes were identified specifically in STGoGS (Table 3, also see Table S7). Among them five genes i.e., brassinosteroid insensitive 1-associated receptor kinase 1 precursor (gnl|UG|Gma#S48313651), brassinosteroid-regulated protein BRU1 (gnl|UG|Gma#S53087048), plasma membrane Ca^2+^-ATPase (gnl|UG|Gma#S5146355), protein kinase (gnl|UG|Gma#S53035243), and RPK (gnl|UG|Gma#S53035182) were up-regulated and two genes i.e., brassinosteroid-regulated protein BRU1 (gnl|UG|Gma#S53087048) and somatic embryogenesis receptor-like kinase-like protein (gnl|UG|Gma#S23066872) were down-regulated. Twelve signaling-related genes differentially expressed under NaCl stress were commonly found in both species. Among them, the expression levels of 10 genes were up-regulated in STGoGS while 9 were up-regulated in SSGoGM only. A leucine-rich repeat hormone-related down-regulated gene in STGoGS was up-regulated in SSGoGM. The change in expression levels across both species was not large.

### Salt responsive candidate genes

Around 136 different genes were annotated which expressed specifically in STGoGS (see Table S8). Among them, 14 genes were stress related (Table 3, also see Table S9). Significantly up-regulated genes were cation diffusion facilitator 9 (gnl|UG|Gma#S53090447), hydrolase (gnl|UG|Gma#S48316466), potassium channel tetramerization domain-containing protein (gnl|UG|Gma#S48316331), protein phosphatase 2C (gnl|UG|Gma#S48316268), abscisic acid 8′-hydroxylase (gnl|UG|Gma#S48312398), multidrug resistance protein ABC transporter (gnl|UG|Gma#S48316693), and stress-related protein (gnl|UG|Gma#S48315849). Significantly down-regulated stress related genes included chitinase class I (gnl|UG|Gma#S5146241), tubulin A (gnl|UG|Gma#S48314982), glutathione S-transferase GST 19 (gnl|UG|Gma#S5146336), putative protease inhibitor (gnl|UG|Gma#S19677361), cytochrome P450 mono-oxygenase CYP81E11 (gnl|UG|Gma#S30676931), tubulin beta-1 chain (gnl|UG|Gma#S48316404), and hypothetical protein (gnl|UG|Gma#S52651928). About 329 genes with unknown functional annotation were found in STGoGS under salt stress. Among these 24 genes were up-regulated and 19 down-regulated in salt stress with a difference in their transcripts per million (TPM) ≥100 from control. Of 151 annotated commonly expressed genes in both species, five belonged to calmodulin, five to glutathione S-transferase and six to the Cytochrome P450 gene families. Among the remaining 135 miscellaneous genes, 37 were related to stresses. About 439 genes with unknown functional annotation were also identified in both species when grown under salt stress. Among them 55 genes appeared as interesting unknown genes present in both species.

The abundance of transcripts encoding translation initiation, elongation factor gamma (EF-G) and elongation factor-1 alpha (EF-1A) factors in the cytoplasm was observed in both the species (Table 4). The transcripts encoding EF-1A factors annotated to the EF-1 complex and mitochondrion was produced stably in STGoGS while expression was reduced in SSGoGM. The expression of three genes (gnl|UG|Gma#S48312868, gnl|UG|Gma#S53090277, and gnl|UG|Gma#S48313669) annotated to elongation factor-1 complex and one gene (gnl|UG|Gma#S48314487) annotated to mitochondrion, and encoding translational elongation factor was stable in STGoGS. Their expression along with another gene (gnl|UG|Gma#S45534682, specific to SSGoGM) was decreased in SSGoGM. An increase in expression was observed for the genes annotated to cytoplasm, translational elongation factor, GTP binding and/or GTPase activity specifically in STGoGS (gnl|UG|Gma#S48315414), in SSGoGM (gnl|UG|Gma#S48314714) and in both (gnl|UG|Gma#S4936922). However, gene gnl|UG|Gma#S45562186 showed significantly reduced expression in SSGoGM but no significant reduction in STGoGS. The expression level of EF-1A-2 (gnl|UG|Gma#S48312404) was significantly increased in SSGoGM. Three genes (gnl|UG|Gma#S52636960, gnl|UG|Gma#S21565950 and gnl|UG|Gma#S48315828) encoding translational initiation factors and one gene (gnl|UG|Gma#S48314567) encoding translational EF-G factor showed increased or stable expression in both species under NaCl stress.

**Table 4:** Translation genes responsive to NaCl stress in salt tolerant genotype of *Glycine soja* (STGoGS) and salt sensitive genotype of *Glycine max* (SSGoGM).

		*Glycine Soja*		*Glycine max*		GO		
Gene	Annotation	LR	PV	LR	PV	Component	Function	Process
**Specifically expressed genes only in ** ***Glycine Soja***								
gnl|UG|Gma#S48315414	T6D22.2 [*At*]	8.9	0.0	-	-	Cytoplasm	TEFA;GTPb	TE
gnl|UG|Gma#S4936922	EF1A [*It*]	1.8	0.0	0.8	0.2	Cytoplasm	TEFA;GTPb;GTPase	TE
**Specifically expressed genes only in both species**								
gnl|UG|Gma#S21565950	Uk [*Mt*]	2.7	0.0	3.3	0.0	-	TIFA	TI;RoT
gnl|UG|Gma#S48315828	Uk [*Gm*]	1.2	0.0	2.4	0.0	-	TIFA	TI
**Specifically expressed genes only in ** ***Glycine max***								
gnl|UG|Gma#S45534682	Uk	-	-	−2.2	0.0	eEF1 complex	TEFA	TE
gnl|UG|Gma#S48314714	EF1A [*Gm*]	-	-	2.2	0.0	Cytoplasm	TEFA;GTPb	TE
gnl|UG|Gma#S23067791	Uk	−0.7	0.1	−1.9	0.0	-	TIFA	TI
gnl|UG|Gma#S48312868	Uk	−0.5	0.0	−1.0	0.0	eEF1 complex	TEFA	TE
gnl|UG|Gma#S53090277	Uk	0.3	0.0	−1.9	0.0	eEF1 complex	TEFA	TE
gnl|UG|Gma#S48314487	Uk	0.2	0.5	−1.9	0.0	Mitochondrion	TEFA;GTPb	TE
gnl|UG|Gma#S52636960	Uk	0.1	0.5	4.8	0.0	-	TIFA	TI
gnl|UG|Gma#S48313669	Uk	−0.7	0.0	−3.0	0.0	eEF1 complex	TEFA	TE
gnl|UG|Gma#S45562186	EF1A, putative [*Rc*]	−0.4	0.1	−2.9	0.0	Cytoplasm	TEFA;GTPb;GTPase	TE
gnl|UG|Gma#S48314567	EF-G [*Gm*]	0.1	0.6	1.5	0.0	Intracellular	TEFA;GTPb;GTPase	TE
gnl|UG|Gma#S48312404	EF1A-2 [*Gh*]	−0.4	0.0	1.8	0.0	Cytoplasm	TEFA;GTPb	TE

LR log2 ratio, PV P-value, EF elongation factor, eEF eukaryotic elongation factor, UK unknown, *At Arabidopsis thaliana*, *It Ignatius tetrasporus*, *Mt Medicago truncatula*, *Gm Glycine max*, *Rc Ricinus communis*, *Gh Gossypium hirsutum*, TEFA translational elongation factor activity, TIFA translational initiation factor activity, TE translational elongation, TI translational initiation, GTPb GTP binding, GTPase GTPase activity.

### Overexpression analysis of salt responsive regulatory genes

**Figure 5:**
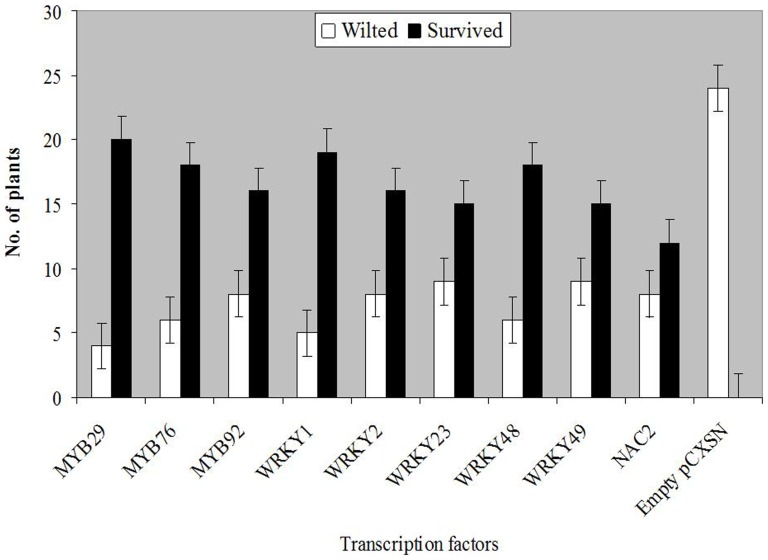
Comparison of over-expression of nine transcription factors (TFs) under 200 mM NaCl stress tested in hairy root system of mosaic soybean plants. On average of two independent repeats, 24 mosaic plants were tested for each TF except GmNAC2 whose 20 mosaic plants were tested. Standard errors are indicated with bars for statistical comparisons. The hairy roots of all survived mosaic plants were PCR positive while wilted one were PCR negative.

**Figure 6:**
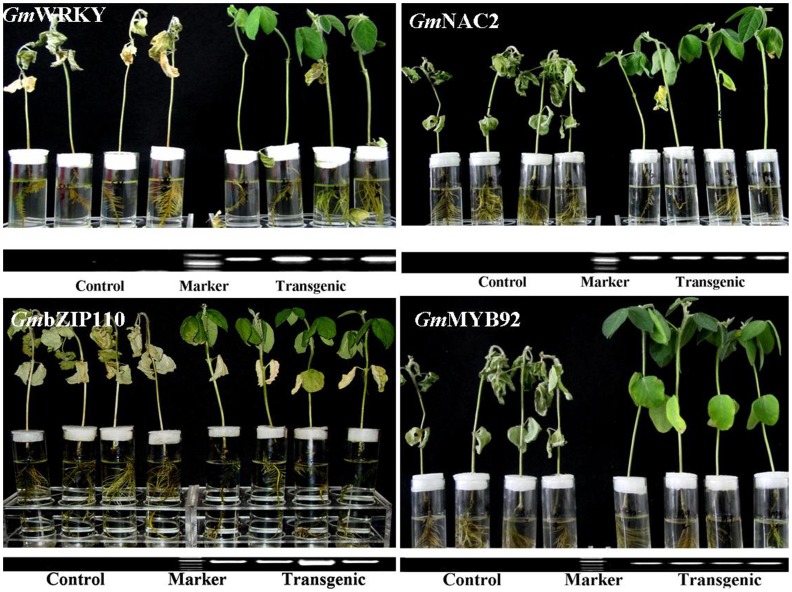
Over-expression analysis of *Gm*WRKY, *Gm*NAC2, *Gm*bZIP110 and *Gm*MYB92 under 200 mM NaCl stress.

The overexpression analysis (OEA) of 13 TFs revealed an increase in salt tolerance of SSGoGM linked to nine TFs which outperformed the control (SSGoGM containing empty vector) in 200 mM NaCl stress (Figure 5). The OEA of four TFs, *Gm*WRKY, GmNAC2, *Gm*bZIP110 and GmMYB92, is presented in Figure 6. PCR analysis confirmed the positive transgenic hairy roots of composite plants only.

### Determination of ABA level

**Figure 7:**
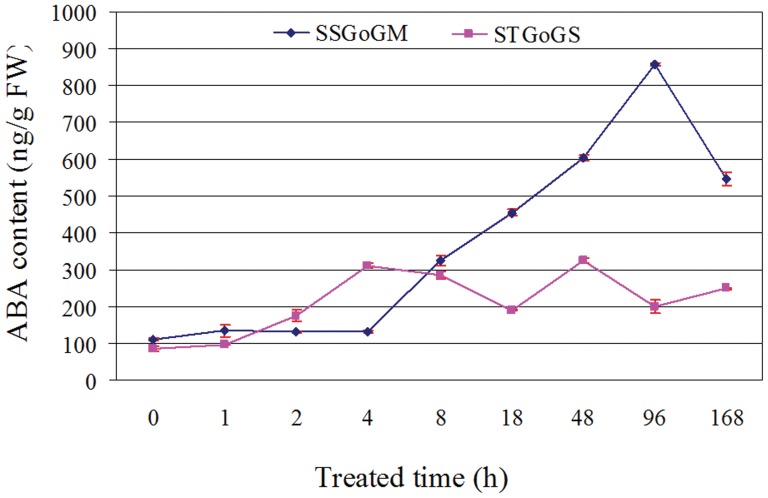
ABA quantification in salt tolerant genotype of *Glycine soja* (STGoGS) and salt sensitive genotype of *Glycine max* (SSGoGM) under 200 mM NaCl stress at various time points.

At the time of 200 mM NaCl stress application (0 h), no significant differences were observed in the ABA levels between the two genotypes (Figure 7). At the 4^th^ h of NaCl stress, the ABA content in STGoGS increased a 2-fold as compared with the SSGoGM. After 8^th^ h to 168^th^ h of NaCl stress, the ABA content in SSGoGM increased a 2 to 4-fold as compared with the STGoGS.

## Discussion

Tag-seq library construction employs Illumina's massively parallel sequencing by synthesis (SBS) protocol [27]. Every sequenced Tag represents a 17-bp sequence tag adjacent to the 3′ NlaIII site of an individual transcript and, therefore, represents a digital count of that transcript. Such counts were recorded from normal and salinity stressed root tissue samples from salt tolerant and sensitive soybean.

The comparison of reference tags, reference genes and clean tags mapped to genes and genomes illustrated no significant deviations in both species (Table 1, also see Table S1). However, there were subtle changes in the pattern. For instance, distinct clean tags were more pronounced in SSGoGM, and their number increased slightly with NaCl stress in both species. The mapping of tags to reference genes in STGoGS was maintained or minutely increased due to NaCl stress while slightly decreasing in SSGoGM. This is further supported by Table S1 where SSGoGM has a greater number of distinct clean tags but a majority (0.193 million out of 0.237 million) have expression levels between 2 and 5 TPM. The expression levels were higher in STGoGS compared to SSGoGM. The number of DEGs was approximately three times in SSGoGM compared to STGoGS but the ratio of up/down-regulated genes was approximately double in STGoGS compared to SSGoGM (Table 2). The analysis of expression profiles suggest that stress tolerance in STGoGS appears to be controlled by genes which are either unique to the species and/or their expression level is stable or higher than SSGoGM (Table 2, see Table S8). Further, 71 genes were up-regulated in STGoGS and down-regulated in SSGoGM, and 35 down-regulated genes in STGoGS were up-regulated in SSGoGM. These are potentially important genes which need further investigation.

The DEGs were dissected across GO terms and pathways in two species (Tables S3, S4, S5, also see Figures S1, S2, S3) and NaCl tolerance was determined in STGoGS. The GO function of two genes gnl|UG|Gma#S53088269 and gnl|UG|Gma#S53086604 described their selective and non-covalent interaction with flavin mononucleotide (FMN), the coenzyme or the prosthetic group of various flavoprotein oxidoreductase enzymes (see Figure S1 and Table S3). These genes play an important role in the electron transport system and oxidation-reduction (redox) reaction. The enhanced expression of FMN binding genes may increase the activities of various enzymes such as oxidoreductases, lipoxygenases and NCED in STGoGS. The former gene was specifically expressed and the later was upregulated in STGoGS while downregulated in SSGoGM. The expression level of genes encoding lipoxygenase and NCED were much higher in STGoGS. The enhanced expression of these genes might increase oxidation of lipids and carotenoids at higher rates in STGoGS compared to SSGoGM.

The NCED catalyzes the cleavage of 9-cis-epoxycarotenoids and converts xanthoxin into abscisic acid (see Figures S1 and S2) [14]. ABA is one of the five classical plant hormones and plays an important role in embryo development, seed dormancy and plant adaptation to various stresses (drought, salinity and cold). ABA is also considered a stress induced hormone. At 4^th^ h of NaCl stress, ABA biosynthesis was high in STGoGS while it started to increase in SSGoGM. ABA-induced stomatal closure represents an important mechanism of plant adaptation to drought. This reaction prioritizes water preservation at a cost of reduced CO_2_ uptake (or less photosynthesis) and reduced mineral transport through the transpiration stream [31]. However, it also helps reduce toxic mineral uptake thus reducing cell mineral toxicity.

In the present studies, the expression pattern of genes involved in translation showed that genes encoding EF-1A factors componential annotated to the elongation factor-1 (EF-1) complex and mitochondrion (Table 4). Previous reports showed the involvement of cytoplasmic eEF-1A in the nuclear export of tRNA in yeast [32] and proteins in mammalian cells via stimulation from the cytoplasmic side of the nuclear envelope without entering the nucleus, thus providing cooperation between the translational apparatus and subcellular trafficking machinery [33]. In the current study, stable or increased expression of translation initiation factors, eEF-1A and eEF-G in the cytoplasm of STGoGS, and eEF-1A-2 in both the species indicates their probable role in mitigating the effect of NaCl stress. The stable or increased expression of eEF-1A from the EF-1 complex and mitochondrion in STGoGS and vice versa in SSGoGM indicates a significant role in conferring salt tolerance. The translational eEF-1A-1 has been shown to interact with phospholipase C, gamma 1 (PLCG1). The protein encoded by this gene catalyzes the formation of inositol 1,4,5-trisphosphate (IP3) and diacylglycerol (DAG) from phosphatidyl inositol 4,5-bisphosphate (PIP2) [19]. This reaction uses calcium as a cofactor and plays an important role in the intracellular transduction of receptor-mediated tyrosine kinase activators. The annotation of gene gnl|UG|Gma#S48315999 showed that it encodes myo-inositol-1-phosphate synthase which catalyzes the synthesis of myo- inositol-1-phosphate that is converted to inositol-1-phosphate (IP1). IP1 is used in a variety of cellular functions including synthesis of IP2 and IP3 [34]. IP3 controls release of Ca^+2^ from the smooth endoplasmic reticulum (ER) or other storage organelles and regulates cell proliferation [35]. Ca^+2^ also plays important roles including stomatal closure, cell division, ion homeostasis, component of cell wall and membranes and in cell signaling. IP3 together with DAG is a secondary messenger molecule used in signal transduction and lipid signaling in biological cells [36].

The class I phosphoinositide 3-kinases (PI3K) indirectly regulates 3-phosphoinositide dependent protein kinase-1 (PDPK1) by phosphorylating PIPs which in turn generates PIP2 and PIP3 [37]. PDPK1 interacts with Na^+^/H^+^ exchange regulatory cofactor 2 (NHERF-2) (gene name SLC9A3R2; also known as Tyrosine kinase activator protein 1) which further interacts with Na^+^/H^+^ antiporter or exchanger 3 (NHE3) or solute carrier family 9 member 3 (SLC9A3) [38]. PDPK1 also interacts with serine/threonine protein kinase (SGK), and activates PI3K. The SGK has been shown to be an important factor in activating certain potassium, sodium, and chloride channels [30].

The expression pattern of gene gnl|UG|Gma#S48312564 was increased in SSGoGM due to salt stress but decreased slightly in STGoGS (see Table S8). This gene is annotated to the catalysis of transposition activity along with regulation of transcription and signal transduction. Transposases are involved in site-specific DNA recombination required for transposition in bacteria and other organisms.

The expression pattern of genes involved in heme binding was high but of those having the additional function of peroxidase was either stable or lower in STGoGS compared to SSGoGM. Most of the genes responsible for ion binding (calcium, magnesium, potassium, zinc, molybdenum, cobalt) were either up-regulated or stable in STGoGS and vice versa in SSGoGM. The heme is responsible for transportation of diatomic gases and electron transfer, and peroxidases are known to play a part in increasing a plant's defenses against pathogens.

The over expression of chitinases has been shown to enhance biotic and abiotic stress tolerance in transgenic tobacco [39], however, enhanced abiotic stress tolerance through inhibition of chitinases has been recently reported in *Arabidopsis* [40]. Clearly the role of chitinases is not well understood and annotations of most chitinases are incomplete. The decreased expression of gene gnl|UG|Gma#S5146241 encoding chitinase class I (involved in the catabolic process of chitin) probably increased chitin level in the cell particularly cell wall and thus might contributed to increased defense response to salinity in STGoGS (see Figure S1 and Table S8). In contrast, up-regulation of genes produced more chitinase class I which might catalyzed and reduced chitin in the cell wall thus contributing to salt sensitivity in SSGoGM.

Transcription factors and signaling regulators are considered to be the most important genes regulating the expression of stress responsive genes [22], [41], [42]. A number of DEGs encoding TFs were identified in STGoGS. TFs are believed to be highly induced by environmental stresses and involved in the regulation of stress responsive genes (see Results). All these groups of TFs identified specifically and/or commonly in the two species showed complicated transcriptional regulatory networks in response to salinity.

Among the signaling-related proteins RPK family proteins are critical components in the mediation of plant responses to stresses [43]. The brassinosteroid insensitive 1 (BRI1)-associated receptor kinase 1 (BAK1) precursor has dual specificity kinase acting on both serine/threonine- and tyrosine-containing substrates and is involved in brassinosteroid (BR) signal transduction. It is involved in programmed cell death (PCD) control and pathogen-associated immunity (PTI). BAK1 positively regulates the brassinosteroid (BR)-dependent plant growth pathway and negatively regulates the BR-independent cell-death pathway [44], [45]. BAK1 is supposed to activate receptor tyrosine kinases (RTKs), serine/threonine protein kinase which in turn activate a master signal transduction gene PDPK1, thus triggering several pathways involved in conferring salt tolerance in STGoGS (Figure 1). The relationship between several signal-related genes expressed in STGoGS (see Table S7) and salt tolerance has not previously been documented. The identification of these genes in STGoGS revealed that various signal molecules act to improve salt tolerance of soybean.

A large number of TFs and signaling related proteins were also identified in both genotypes which have comparable expressions. Most of these were annotated to the plant pathogen interaction pathway (see Figure S4) and are thus involved in conferring salt tolerance. The hormone, light, and pathogenesis signaling-related genes were also found to be DE in both genotypes, indicating that multiple signal regulation exists in soybean under salinity stress as earlier reported in tomato under drought stress [9].

The current study also presents insight into some important salt responsive genes in addition to the above narrated genes of translation, ABA biosynthesis, Chitinases etc. These genes were annotated in ATP-binding cassette (ABC) transporters and plant pathogen interaction and related pathways like endocytosis and ubiquitin mediated proteolysis.

The plants of SSGoGM containing over-expressed TFs survived in solution culture containing 200 mM NaCl whereas the control did not (Figure 6). Interestingly, all lower leaves of plants containing *Gm*bZIP110 TF and those of the control plants remained intact while the majority of plants containing *Gm*WRKY and *Gm*NAC2 TFs shed their lower leaves. The lower leaves were green and intact in *Gm*MYB plants. These TFs need further exploration to get insight in their involvement in different processes and pathways, and their interaction with other genes to confer salt tolerance.

It is concluded that the control of salinity tolerance in soybean is clearly complex. In the current study we identified a series of genes and transcription factors that are potentially important in conferring salinity tolerance in soybean. The gene expression levels and ratio of up/down-regulated genes was greater in tolerant plants. The overexpression of nine TFs in soybean hairy root system enhanced salt tolerance in SSGoGM. Nevertheless, genome-wide expression studies of this nature need to be implemented together with soybean-breeding programs. This will require access to high-throughput screening platforms if new salt tolerant soybean varieties are to be developed.

## Materials and Methods

### Ethics statement

No specific permits were required for the described studies, as it only included collection of seed of wild soybean species which is not endangered or protected species in China.

### Plant materials and growing conditions

The seeds of a salt tolerant wild genotype of *Glycine soja* (STGoGS) were collected from naturally growing plants on the Chinese coast of Yellow Sea. The seeds of a salt sensitive genotype of *Glycine max* cv ‘Market No. 1’ (SSGoGM) were purchased from a local market. The two species were grown in pots and treated with 200 mM NaCl solution for seven days to test their response to salinity (Figure 1A). The SSGoGM was further tested in two sets of glasstubes (Figure 1C): one set was treated with 200 mM NaCl and the other was control (Figure 1D). Two weeks old seedlings from seeds of the respective species were divided into two sets. One set was treated with 200 mM NaCl and the other with water containing no added salt. Both sets from each species were kept overnight in a growth chamber set at 24°C (see Supplementary Methods S1 for details of plant growing conditions).

### Total RNA isolation, reverse transcription, Tag library construction and sequencing

Total RNA was isolated from control and salinity treated soybean roots separately. Sample preparation and sequencing was conducted using an Illumina gene expression sample prep kit and Solexa sequencing chip (flowcell) following the manufacturer's instructions. Total RNA extract (6 µg) was treated with oligo (dT) magnetic beads adsorption to purify mRNA, and then oligo (dT) was used for reverse transcription polymerase chain reaction (RT-PCR) to synthesize double-stranded cDNA (Figure 2).

A restriction enzyme NlaIII was used to digest cDNA which recognizes and cuts CATG sites on cDNA (Figure 2). The magnetic beads precipitation was used to purify cDNA fragments with 3′ ends and Illumina adapter 1 was added to their 5′ ends. The junction of Illumina adapter 1 and CATG site is the recognition site of MmeI, which is a type of endonuclease with separated recognition sites and digestion sites. It cuts at 17 bp downstream of the CATG site, producing tags with adapter 1. After removing 3′ fragments with magnetic beads precipitation, Illumina adapter 2 was introduced at 3′ ends of tags, acquiring tags with different adapters at both ends to form a tag library. After 15 cycles of linear PCR amplification: 98°C for 30 sec, followed by 15 cycles of 98°C for 10 sec, 60°C for 30 sec and 72°C for 15 sec, and then 72°C for 5 min, 85 base strips were purified by 6% TBE polyacrylamide gel electrophoresis (PAGE). These strips were then digested, and the single-chain molecules were fixed onto the Solexa sequencing chip (flowcell). Each molecule was grown into a single-molecule cluster sequencing template through Situ amplification. Then four types of color labeled nucleotides were added and sequencing by the synthesis (SBS) method was performed. Each tunnel generated millions of raw reads with a sequencing length of 35 bp.

### Data analysis

Raw sequence data were cleaned from low quality sequences and impurities. Tag libraries construction, sequence saturation, reproducibility and filtration for unambiguous tags were adopted. The number of unambiguous clean tags for each gene was calculated and then normalized to number of transcripts per million clean tags (TPM) [27], . A rigorous algorithm was developed to identify differentially expressed genes (DEGs) among the control and NaCl treated samples [47]. The sense-antisense transcripts were annotated for expression and detection of new transcripts. The cluster analysis of gene expression patterns was performed with “cluster” [48] and “JavaTreeview” (Saldanha 2004) softwares. The differentially expressed genes were analyzed for gene ontology (GO) terms and pathways enrichments (see Supplementary Methods S1 for details).

### RT-PCR, qPCR and overexpression analyses

Total RNAs isolated, using TRIzol® reagent (Invitrogen & Co.) following the manufacturer's instructions, from roots of STGoGS and SSGoGM under 200 mM NaCl stress and control conditions were used for RT-PCR analysis. Sample preparation and qPCR analysis was conducted following SYBR® Premix Ex Taq™ (Perfect Real Time) in a Roche LightCycler 2.0, using LightCycler software (build 4.1.1.21), (LightCycler® Carousel-based System, F. Hoffmann-La Roche Ltd, Germany). Phosphoenolpyruvate carboxylase (PEPC) was used as the internal control [49] (see Supplementary Methods S1 for details).

Four TFs, one from each of *Gm*WRKY, *Gm*NAC, *Gm*bZIP and *Gm*MYB TF gene families, were selected for presentation on the basis of DGEP data and overexpression analysis out of 13 tested TFs. These TFs were cloned using pCXSN vector [50] containing double tobacco mosaic virus promoter 35S and transformed in *Agrobacterium rhizogenes*. An *Agrobacterium* mediated soybean transformation system to develop hairy roots (mosaic plant) was used for overexpression analysis (OEA) of four TFs following a modified method from earlier report [51]. The seeds of SSGoGM were sown in pots to develop seedlings following plant growing conditions described in Supplementary Methods S1. One day before injection, *Agrobacterium* carrying gene of interest was grown overnight in LB medium containing kanamycin at 28°C and shaking @180 rpm. The transformed *Agrobacterium* was harvested by centrifugation at 5000 rpm for two mins at room temperature. The pellet was suspended gently in 10 mM MgCl_2_ solution followed by two washings. The OD600 of final suspension was adjusted to 0.6. On emergence of true leaves, 5–10 µl inoculum of transformed *Agrobacterium* was injected at juncture of two cotyledons. One TF was injected in around 100 seedlings. The inject point was covered with soil. Water was applied as per requirement. Around 10–15 days after injection, hairy roots developed at inject point in more than 60% of the seedlings. The true roots were cut-off below the inject point where hairy roots developed, only one hairy root was kept by cutting all others and seedlings were hold in the glass-tubes containing ½ strength Hoagland solution as shown in Figure 6. The tubes holding mosaic seedlings were placed in growth chamber set at day/night temperatures of 28/25±2°C and photoperiod of 12 h. After one week of growth in the growth chamber, the control and transformed mosaic soybean seedlings were treated with 200 mM NaCl and evaluated on 7^th^ day of stress.

### ABA quantification

The root samples of STGoGS and SSGoGM were taken at 0, 1, 2, 4, 8, 18, 48, 96 and 168 hours of 200 mM NaCl treatment, placed immediately in liquid nitrogen and stored in an ultrafreezer (−80°C). The samples were grinded and ABA content was quantified following procedure given elsewhere [52].

## Supporting Information

Methods S1 **Supplementary materials and methods.**
(DOC)

Figure S1 **Molecular functions enriched with differentially expressed genes specific to salt tolerant genotype of** ***Glycine soja*** **(STGoGS), salt sensitive genotype of** ***Glycine max*** **(SSGoGM) and common in both.**
(TIFF)

Figure S2 **Biological processes enriched with differentially expressed genes specific to salt tolerant genotype of** ***Glycine soja*** **(STGoGS), salt sensitive genotype of** ***Glycine max*** **(SSGoGM) and common in both.**
(TIFF)

Figure S3 **Cellular components enriched with differentially expressed genes specific to salt tolerant genotype of** ***Glycine soja*** **(STGoGS), salt sensitive genotype of** ***Glycine max*** **(SSGoGM) and common in both.**
(TIFF)

Figure S4 **Salt responsive genes from salt tolerant genotype of** ***Glycine soja*** **(STGoGS) annotated to plant pathogen interaction.**
(TIFF)

Table S1 **Summary results of reference tag database, tag libraries and tag mapping to genes and genomes from** ***Glycine soja*** **and** ***Glycine max*** **in presence and absence of NaCl stress.**
(XLS)

Table S2 **Specifically differentially expressed genes in salt tolerant genotype of** ***Glycine soja*** **(STGoGS), salt sensitive genotype of** ***Glycine max*** **(SSGoGM) and in both species.**
(XLS)

Table S3 **Molecular functional based classification of salt responsive genes.**
(XLS)

Table S4 **Biological process-wise classification of salt responsive genes.**
(XLS)

Table S5 **Cellular component classification of salt responsive genes.**
(XLS)

Table S6 **Pathway annotation of salt responsive genes.**
(XLS)

Table S7 **Expression level of selected transcription factors and signaling genes responsive to salinity stress in salt tolerant genotype of** ***Glycine soja*** **(STGoGS) and salt sensitive genotype of** ***Glycine max*** **(SSGoGM).**
(XLS)

Table S8 **Expression level of all significant transcription factors, signaling genes and structural genes responsive to salinity stress in salt tolerant genotype of** ***Glycine soja*** **(STGoGS) and salt sensitive genotype of** ***Glycine max*** **(SSGoGM).**
(XLS)

Table S9 **Salt responsive genes in salt tolerant genotype of** ***Glycine soja*** **(STGoGS) and salt sensitive genotype of** ***Glycine max*** **(SSGoGM).**
(XLS)
