# Supplementary material for: Uncovering the Salt Response of Soybean by Unraveling Its Wild and Cultivated Functional Genomes Using Tag Sequencing
Source: PLoS One. 2012 Nov 28;7(11):e48819. doi: 10.1371/journal.pone.0048819 (PMC3509101; doi:10.1371/journal.pone.0048819)
Supplement: Methods S1 — Supplementary materials and methods. (DOC) [file pone.0048819.s001.doc]

# Uncovering the salt response of soybean by unraveling its wild and cultivated functional genomes

**Supplementary materials and methods**

**Plant growing conditions**

Around 20 seeds of each species were grown in the greenhouse in separate pots of size 22 cm dia containing soil and compost in a 3:2 ratio. The day/night temperatures were 28/25 ± 2 ºC and photoperiod of 12 h. Water was applied as per requirement. Two weeks old plants were up-rooted from each pot and placed in two glass beakers. One beaker contained 200 mM NaCl solution while second water with no added salt.

**Bioinformatics analyses for quality evaluation**

***Raw sequence data processing and cleaning dirty tags***

Sequencing-received raw image data is transformed by base calling into sequence data, which is called raw data or raw reads, is stored in FASTQ format. This type of files is that the client gets, in which stores reads sequences and quality. Each read is described in four lines in FSATQ files.

@HWI-EAS80_4_4_1_554_126

GTATGCCGTCTTCTGCTTGAAAAAAAAAAACATAAAACAA

+HWI-EAS80_4_4_1_554_126

hhhhhhhhhhhhhhhhhhh[hEhSJPLeLdCLEN>IXHAA

The line 1 and 3 are sequences name generated by the sequence analyzer; line 2 is sequence; line 4 is sequencing quality value, in which every letter corresponds to a base in line 2; the base's sequencing quality is the ASCII value that the letter in line 4 refers to minus 64. For example, the ASCII of h is 104, so the corresponding sequencing quality value is 40. Sequencing quality value ranges of Solexa bases are from 0 to 40.

Since tags are only 21 nt long while the sequencing reads are 35 nt long. Raw sequences have 3' adaptor sequences/fragments as well as a few low quality sequences and several types of impurities. Raw sequences were transformed into clean tags after following certain steps of data processing, and clean tags data were generated after filtering dirty tags from raw data.

Data-processing steps;

1. Removal of 3' adaptor sequence.
2. Removal of empty reads (only 3' adaptor sequences but no tags)
3. Removal of low quality Tags (Tags with unknown sequences 'N')
4. Removal of Tags which are too long or too short, leaving Tags of 21nt long
5. Generate Clean Tags

***Sequencing saturation and experimental reproducibility analyses***

The saturation analysis was performed to check whether the number of detected genes keeps increasing when sequencing amount (total tag number) increases. When sequencing amount reached 2M or higher, the number of detected genes almost ceased to increase. Two parallel experiments were executed for the reliability of experimental results as well as operational stability. Correlation analysis between the two experiments was done (data not presented).

***Standardization or normalization of gene expression data***

A preprocessed database of all possible CATG+17-nt tag sequences was created, using reference gene sequences of soybean and closely related species (if there were no reference gene sequences for soybean). All clean tags were mapped to the reference sequences. Clean tags mapped to reference sequences from multiple genes were filtered. Remainder clean tags were designed as unambiguous clean tags. The number of unambiguous clean tags for each gene was calculated and then normalized to TPM (number of transcripts per million clean tags) .

***Identification of differentially expressed genes***

A rigorous algorithm was developed to identify differentially expressed genes (DEGs) among the control, and NaCl treated samples . Denote the number of unambiguous clean tag from gene A as x, as every gene's expression occupies only a small part of the library, the p(x) is in the Poisson distribution.


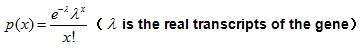


The total clean tag number of the sample 1 is N 1, and total clean tag number of sample 2 is N 2; gene A holds x tags in sample 1 and y tags in sample 2. The probability of gene A expressed equally between two samples can be calculated with:


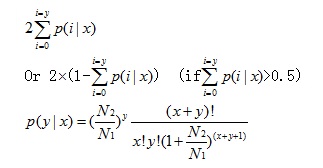


P value corresponds to differential gene expression test and FDR (False Discovery Rate) was used to determine its threshold . We used "FDR≤0.001 and the absolute value of log_2_ratio≥1" as the threshold to judge the significance of gene expression difference.

***Expression annotation of sense-antisense transcripts and detection of new transcripts***

Virtual sense and antisense tag sequence databases were generated. Briefly, all NlaIII sites were identified for each sequence, and the adjoining 17 bp in the 3’ direction were designated the sense tags, while the 17 bp in the 5’ direction were designated the antisense tags. Comparing with microarray, DGEP detects new transcripts without pre-designed probes. The mapped clean tags that could not be mapped to mRNA, mitochondria and chloroplast to the whole soybean genome, provided unique transcripts mapped to those tags.

***Clustering analysis of differential gene expression pattern***

The cluster analysis of gene expression patterns was performed with "cluster" and "javaTreeview" softwares. In Figure 4, each column represents an experimental condition, each row represents a gene. Red to green color indicates high to low expression levels.

**Gene ontology functional enrichment analysis for DEGs**

Using gene ontology (GO) enrichment analysis, to comprehensively describe molecular function, cellular component and biological process of genes and their products, all DEGs were mapped to GO-terms in GO database, looking for significantly enriched GO terms in DEGs comparing to the genome background. The calculating formula is:


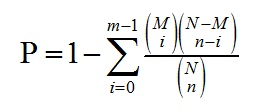


Where N is the number of all genes with GO annotation; n is the number of DEGs in N; M is the number of all genes that are annotated to the certain GO terms; m is the number of DEGs in M.

**Pathway enrichment analysis for DEGs**

Different genes usually cooperate with each other to exercise their biological functions. Pathway-based analysis helps to further understand genes biological functions. Kyoto Encyclopedia of Genes and Genomes (KEGG) is the major public pathway-related database. Significantly enriched metabolic pathways or signal transduction pathways in DEGs were identified via pathway enrichment analysis using KEGG, public pathway-related database, and comparing with the whole genome background. The calculating formula was the same as that in GO analysis. Here N is the number of all genes that with KEGG annotation, n is the number of DEGs in N, M is the number of all genes annotated to specific pathways, and m is number of DEGs in M.

**RT-PCR**

The cDNAs were synthesized from 2.5 μg total RNA using the PrimeScript^TM^ RTase (TaKaRa Bio Inc**; Shiga, Japan** <http://www.takara-bio.com/>) following the manufacturer’s instructions. In first step, a total mix of 20 μl was prepared using 16 μl of total RNA, 2 μl of 100 μM oligo (dt) and 2 μl of 10 mM dNTP. The mix was incubated at 65°C for 5 min followed by ice bath for > 2 min. In second step, a total volume of 20 μl was prepared using 8 μl of 5× buffer, 2 μl of PrimeScript^TM^ RTase, 1 μl of RNase Inhibitor and 9 μl of RNase free water, and added into micro-tube containing product from first step. Reverse transcription **was conducted in a thermo-cycler (LabCycler, SensoQuest Biomedizinische Elektronik,GmbH., Göttingen, Germany) set with temperature of** 42 °C for 90 min and 72 °C for 10 min.

**qPCR analysis**

Primers for qPCR were designed with the **Primer Premier5.0** program (<http://www.premierbiosoft.com/crm/jsp/com/pbi/crm/clientside/ProductList.jsp>). Sample preparation and qPCR analysis was conducted following SYBR® Premix Ex Taq™ (Perfect Real Time). A 10 μl of mix consisted of 5 μl of SYBR® Premix Ex Taq™ (TaKaRa Bio Inc**; Shiga, Japan** <http://www.takara-bio.com/>), 0.8 μl of each primer (forward and reverse), 1 μl of template (cDNA) and 2.4 μl of d_2_H_2_O. The qPCR was conducted in a Roche LightCycler 2.0, using LightCycler softwar (build 4.1.1.21), (LightCycler® Carousel-based System, F. Hoffmann-La Roche Ltd, Germany). The software used the following cycling parameters: a hot start at 95°C for 30 sec, 45 PCR cycles of quantification consisted of 95°C for 5 sec, 58°C for 10 sec and 72°C for 20 sec followed by melting with melting curve conditions of 95 °C for 5 sec, 60°C for 5 sec, 95°C and 45°C for 5 sec. Phosphoenolpyruvate carboxylase (PEPC) was used as the internal control .

**References**

1. t Hoen PAC, Ariyurek Y, Thygesen HH, Vreugdenhil E, Vossen RHAM, et al. (2008) Deep sequencing-based expression analysis shows major advances in robustness, resolution and inter-lab portability over five microarray platforms. Nucleic Acids Research 36: e141-e141.

2. Morrissy AS, Morin RD, Delaney A, Zeng T, McDonald H, et al. (2009) Next-generation tag sequencing for cancer gene expression profiling. Genome Research 19: 1825-1835.

3. Audic S, Claverie M (1997) The significance of digital gene expression profiles. Genome Research 7: 10.

4. Benjamini Y, Yekutieli D (2001) The control of the false discovery rate in multiple testing under dependency. The Annals of Statistics 25: 24.

5. Eisen MB, Spellman PT, Brown PO, Botstein D (1998) Cluster analysis and display of genome-wide expression patterns. Proceedings of the National Academy of Sciences 95: 6.

6. Saldanha AJ (2004) Java Treeview--extensible visualization of microarray data. Bioinformatics 20: 3246-3248.

7. Tuteja JH, Clough SJ, Chan W-C, Vodkin LO (2004) Tissue-specific gene silencing mediated by a naturally occurring chalcone synthase gene cluster in *Glycine max* The Plant Cell 16: 819-835.
