# Supplementary figures and images for: Uncovering the Salt Response of Soybean by Unraveling Its Wild and Cultivated Functional Genomes Using Tag Sequencing
Source: PLoS One. 2012 Nov 28;7(11):e48819. doi: 10.1371/journal.pone.0048819 (PMC3509101; doi:10.1371/journal.pone.0048819)

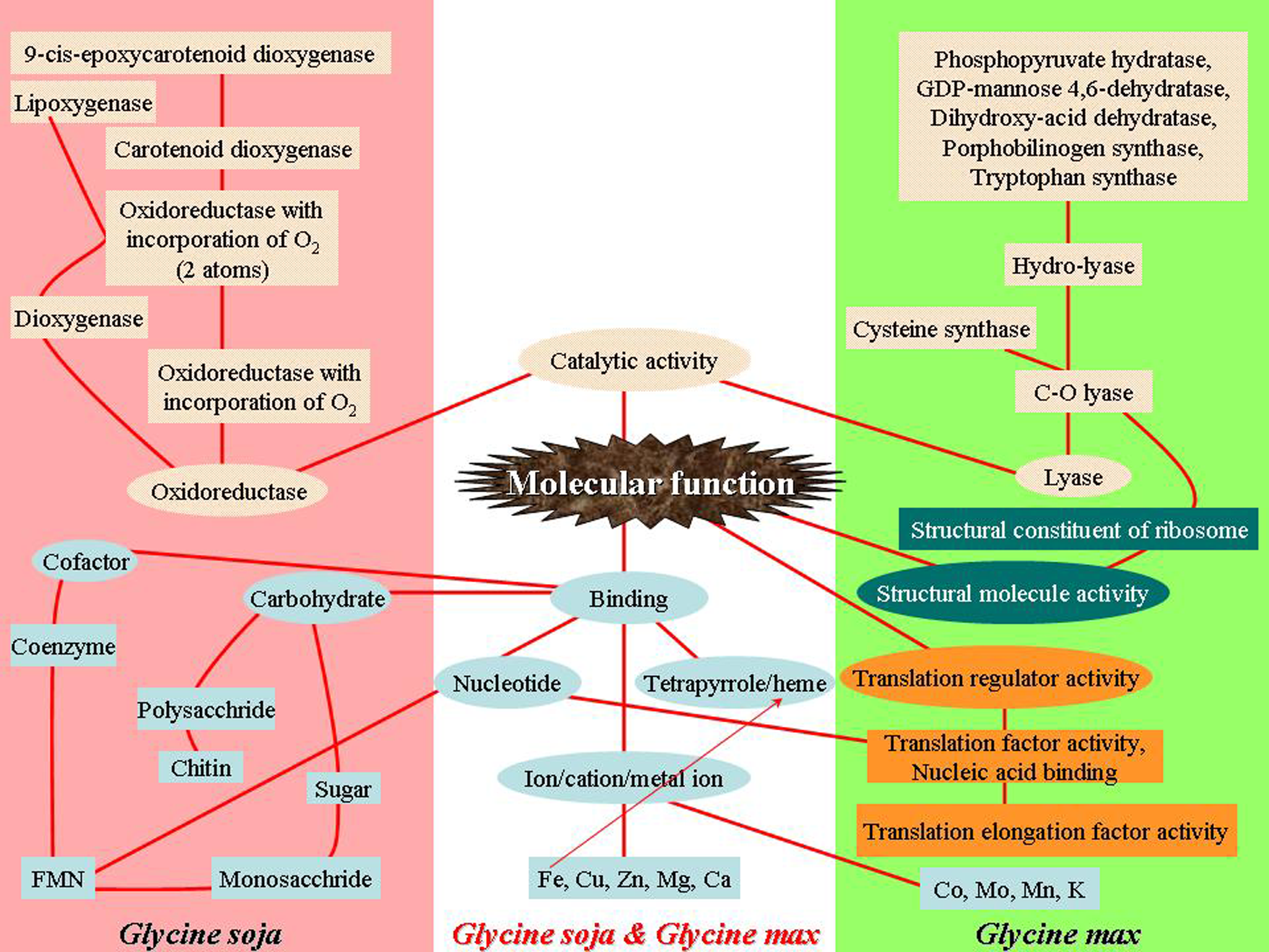

Supplement: Figure S1 — Molecular functions enriched with differentially expressed genes specific to salt tolerant genotype of Glycine soja (STGoGS), salt sensitive genotype of Glycine max (SSGoGM) and common in both. (TIFF) [file pone.0048819.s002.tiff]

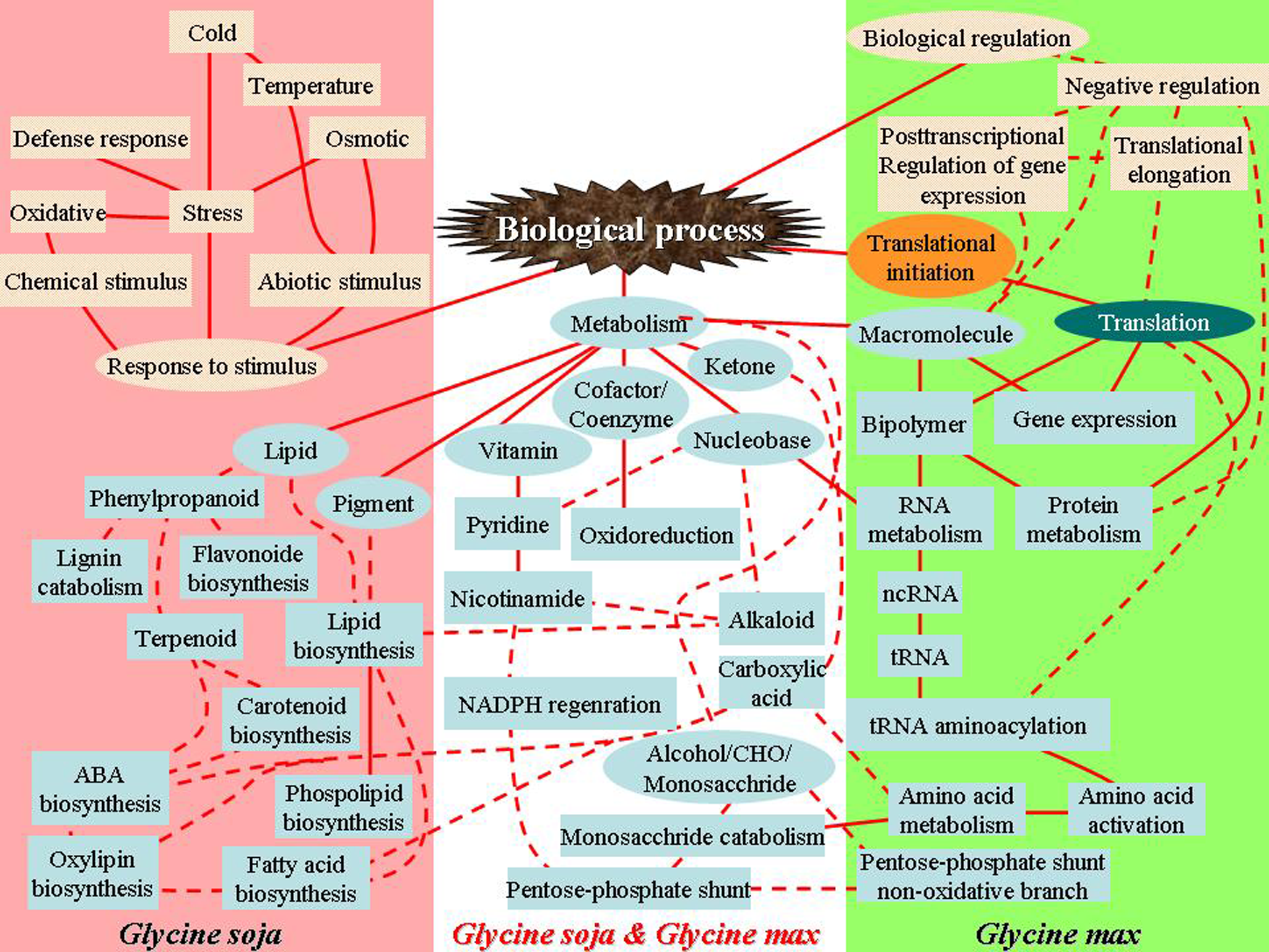

Supplement: Figure S2 — Biological processes enriched with differentially expressed genes specific to salt tolerant genotype of Glycine soja (STGoGS), salt sensitive genotype of Glycine max (SSGoGM) and common in both. (TIFF) [file pone.0048819.s003.tiff]

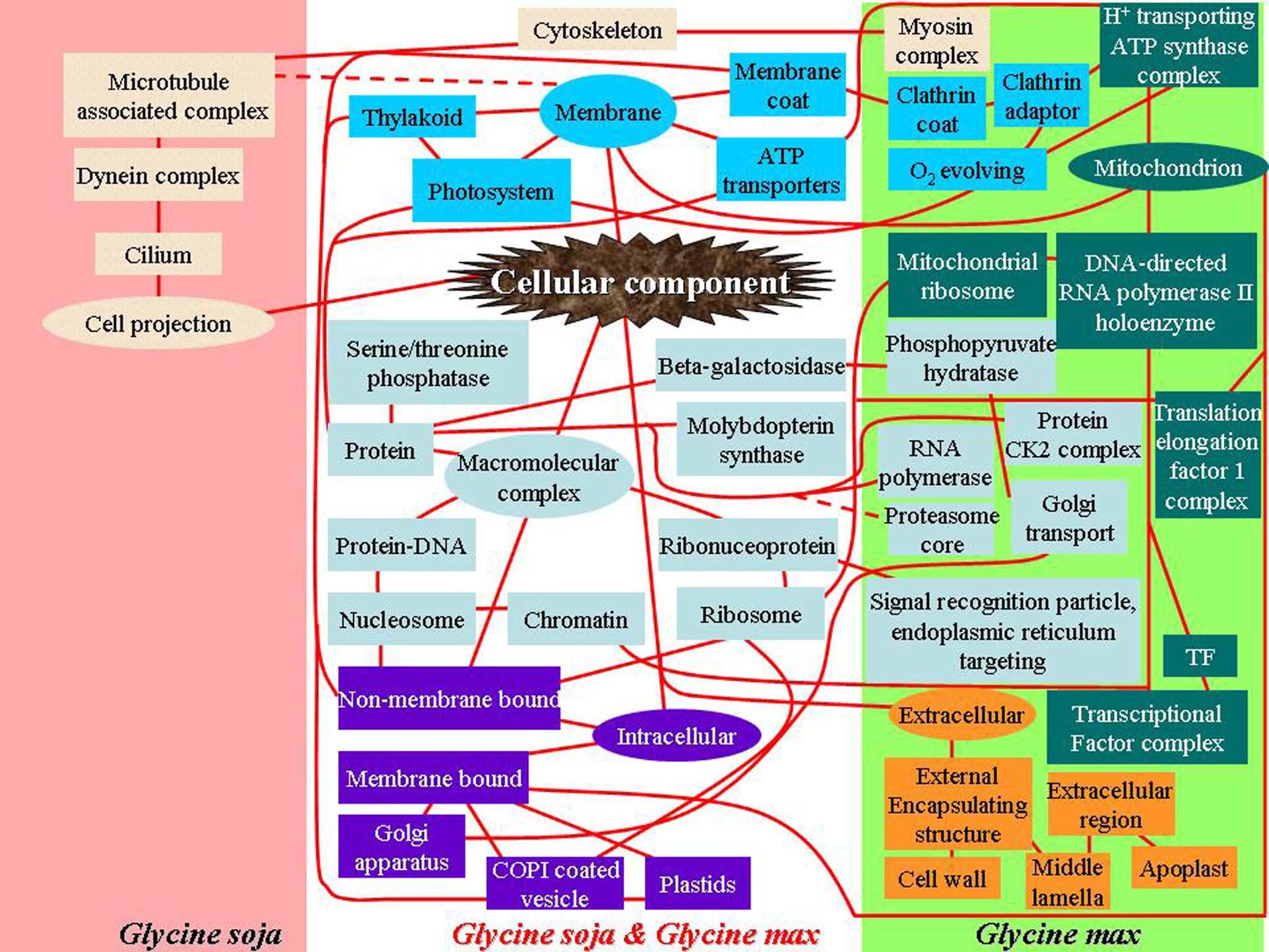

Supplement: Figure S3 — Cellular components enriched with differentially expressed genes specific to salt tolerant genotype of Glycine soja (STGoGS), salt sensitive genotype of Glycine max (SSGoGM) and common in both. (TIFF) [file pone.0048819.s004.tiff]

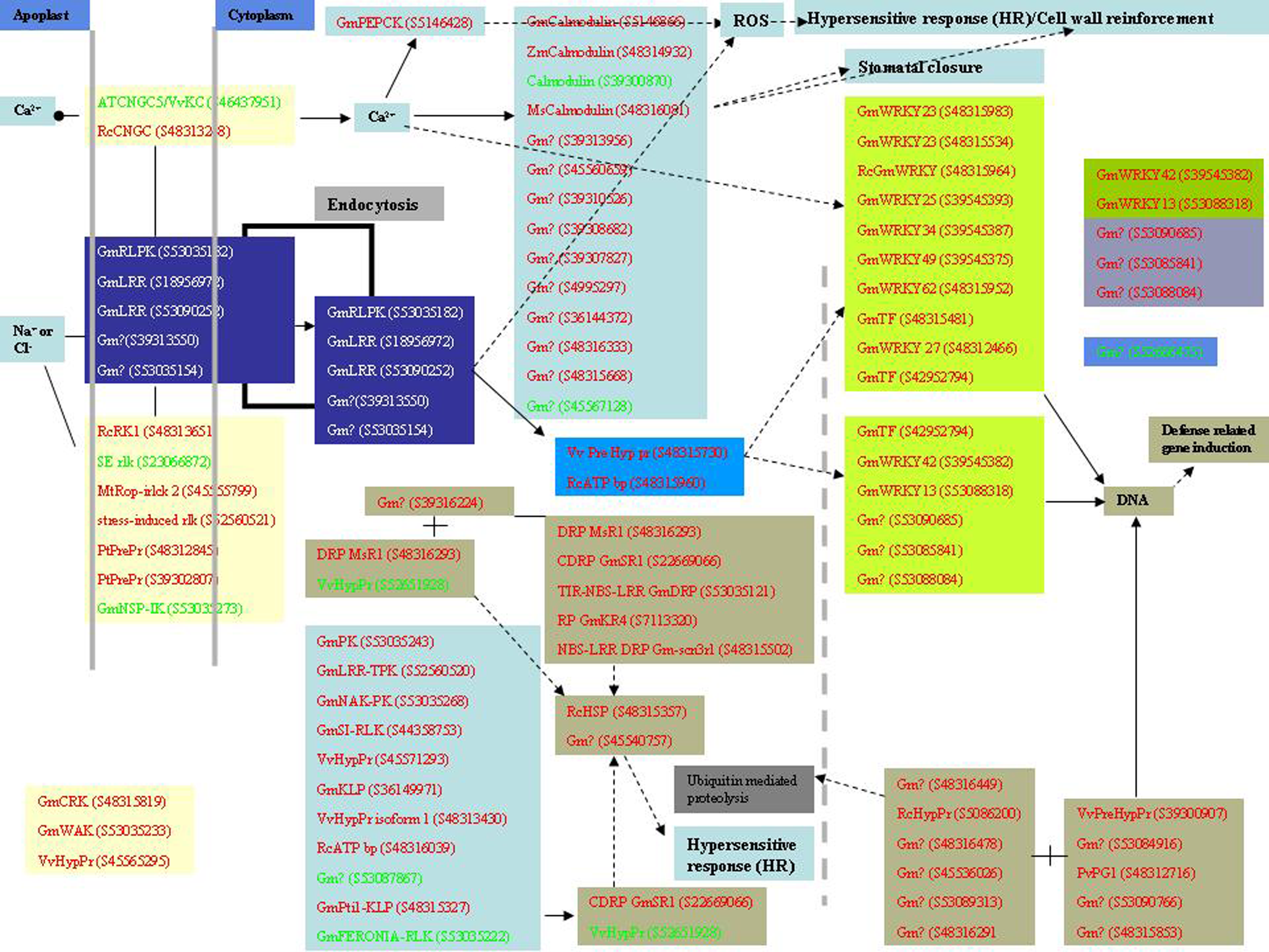

Supplement: Figure S4 — Salt responsive genes from salt tolerant genotype of Glycine soja (STGoGS) annotated to plant pathogen interaction. (TIFF) [file pone.0048819.s005.tiff]
